# Evaluation of a genetic risk score for severity of COVID-19 using human chromosomal-scale length variation

**DOI:** 10.1186/s40246-020-00288-y

**Published:** 2020-10-09

**Authors:** Christopher Toh, James P. Brody

**Affiliations:** Department of Biomedical Engineering, University of California, Irvine, USA

**Keywords:** COVID-19, Genetic risk score, UK biobank, Machine learning

## Abstract

**Introduction:**

The course of COVID-19 varies from asymptomatic to severe in patients. The basis for this range in symptoms is unknown. One possibility is that genetic variation is partly responsible for the highly variable response. We evaluated how well a genetic risk score based on chromosomal-scale length variation and machine learning classification algorithms could predict severity of response to SARS-CoV-2 infection.

**Methods:**

We compared 981 patients from the UK Biobank dataset who had a severe reaction to SARS-CoV-2 infection before 27 April 2020 to a similar number of age-matched patients drawn for the general UK Biobank population. For each patient, we built a profile of 88 numbers characterizing the chromosomal-scale length variability of their germ line DNA. Each number represented one quarter of the 22 autosomes. We used the machine learning algorithm XGBoost to build a classifier that could predict whether a person would have a severe reaction to COVID-19 based only on their 88-number classification.

**Results:**

We found that the XGBoost classifier could differentiate between the two classes at a significant level (*p* = 2 · 10^−11^) as measured against a randomized control and (*p* = 3 · 10^−14^) as measured against the expected value of a random guessing algorithm (AUC = 0.5). However, we found that the AUC of the classifier was only 0.51, too low for a clinically useful test.

**Conclusion:**

Genetics play a role in the severity of COVID-19, but we cannot yet develop a useful genetic test to predict severity.

## Introduction

The course of COVID-19 varies from asymptomatic to severe (acute respiratory distress, cytokine storms, and death) in patients. The basis for this range in symptoms is unknown. One possibility is that genetic variation is partly responsible for the highly variable response to infection.

Human genetic variation can affect susceptibility and resistance to viral infections [1]. For instance, variants in the gene IFITM3 affect the severity of seasonal influenza [2]. Patients hospitalized from seasonal influenza had a particular allele of the gene IFITM3 at a higher rate than expected from the general population. Laboratory work determined that this particular allele can alter the course of the influenza virus infection.

We have previously shown that chromosomal-scale length variation is a powerful tool to analyze genome-wide associations [3]. This method is particularly appealing for genetic risk scores because it includes epistatic effects that might be missed with conventional genome-wide association studies. Others have used machine learning in combination with copy number variation to predict cancer risk [4].

The purpose of this paper is to evaluate how well a genetic risk score based on chromosomal-scale length variation and machine learning classification algorithms can predict severity of response to SARS-CoV-2 infection. We evaluated this approach on a dataset of 931 patients who had a severe reaction to COVID-19 in the early part of the 2020 global pandemic. These patients had been previously genotyped as part of the UK Biobank.

## Methods

Data was obtained from the UK Biobank under Application Number 47850. First, we downloaded the “l2r” files from the UK Biobank. Each chromosome has a separate “l2r” file. Each “l2r” file contained 488,377 columns and a variable number of rows. Each column represented a unique patient in the dataset, who is only identified by an encoded identification number. Each row represented a measurement at a different location in the genome. The values in the file represent the log (base 2) of the ratio of measured intensity measured in a microarray relative to the expected two copies at that location in the genome.

After downloading the “l2r” data from the UK Biobank, we computed the mean l2r value for a portion, we chose 25%, of the chromosome for each patient in the dataset. This process produced a dataset where each person was represented by a series of 88 numbers. Each number represents the length variation for 25% of the 22 non-sex chromosomes. A value of 0 (log_2_ ration) represents the nominal average length of that portion of the particular chromosome. We call this dataset the chromosomal-scale length variation (CSLV) dataset.

This CSLV dataset was matched with the UK Biobank COVID-19 dataset. The COVID-19 data were provided to UK Biobank by Public Health England. UK Biobank matched the person in the Public Health England data with UK Biobank’s internal records to produce the person’s encoded participant identification number. The dataset we have provided by UK Biobank contains the participant ID, date the specimen was taken, laboratory that processed the sample, whether the patient was an inpatient when the sample was taken, and the result (positive/negative) of the test. The UK Biobank continues to update the data approximately biweekly.

The criteria for testing and interpretation of results in the UK Biobank COVID-19 data has evolved. A positive test in this dataset earlier than 27 April 2020 was a good indication that the person had severe disease. During this initial period of the pandemic, SARS-CoV-2 testing was only performed on symptomatic people and this particular dataset only includes people tested in a hospital. After 27 April 2020, NHS instructed hospitals to test all non-elective patients admitted, including asymptomatic patients. The UK Biobank dataset released after 27 May 2020 includes “pillar 2” positive test results. These “pillar 2” tests include people in hospitals for non-elective procedures and staff screening. These results can include asymptomatic patients.

Using the CSLV-COVID-19 dataset, we selected all people who tested positive before 27 April 2020 and labeled these as people having a severe reaction to COVID-19. We segmented these into three overlapping datasets, as shown in Table 1. We constructed an age-matched control group of the same size that had an identical age profile as those in the severe reaction group. The age-matched control group was selected from the entire UK Biobank dataset, excepting those few who had a severe reaction to COVID-19. Since only a small fraction of the people in the UK Biobank had a severe reaction to COVID-19, we could rerun the analysis with a different age-matched control group many times to build up statistics. We chose this method of selecting the control group based on the finding that severe reactions to COVID-19 are both a strong function of age and uncommon (only about 20% of those infected with SARS-CoV-2 require ICU admission even among those in their 70s) [5, 6].

**Table 1:** We segmented the dataset into three overlapping subsets. The first, which we called “1930” contained all UK Biobank participants born after 1930 who had a severe reaction to SARS-CoV-2 infection before 27 April 2020. The two subsets contained people born after 1940 and after 1950

Dataset	Number
1930 (< 90 years of age)	981
1940 (< 80 years of age)	880
1950 (< 70 years of age)	468

We used the H2O machine learning package in R to create XGBoos t[7] models that were trained to classify a person in the dataset, consisting of those who had a severe reaction and age-matched controls, based solely on their chromosomal-scale length variation data.

## Results

**Fig. 1:**
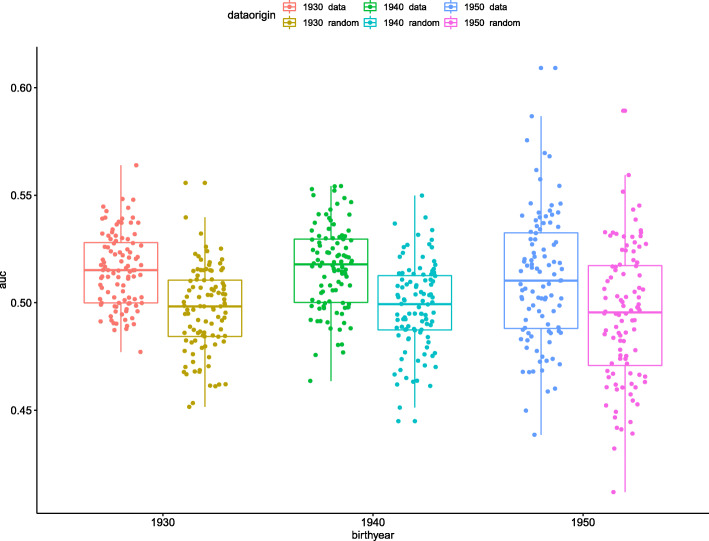
This boxplot figure presents the results of the machine learning predictions. We created three different datasets, one which includes all patients less than 90 years old, the second includes every patient less than 80 years old, and the third with every patient less than 70 years old. These are indicated as the oldest birthyear “data.” Each dataset included an equal number of patients with a “severe reaction” to COVID-19 and an equal number of age-matched people drawn from the general UK Biobank population, “normal.” For comparison, we took those three datasets and randomly permuted the status (“severe reaction” or “normal”) and repeated the process. This randomly permuted dataset is labeled oldest birthyear “random.” For each dataset, we repeated the whole process 100 times, each time with a different set of age-matched people from the general UK Biobank population

The results are presented in Fig. 1 and Table 2. As Fig. 1 shows, we found a significant difference between all three age groupings and their corresponding random controls. This finding indicates that germ line genetics of the infected patient, as represented by the set of chromosomal-scale length variation numbers, is correlated with the severity of COVID-19.

**Table 2:** We compared the difference in mean AUC values between the various datasets using a *t* test. The datasets consisting of people born after 1930, 1940, and 1950 all showed significant differences with the corresponding random control. Those three datasets also showed significant differences between the mean AUC and 0.5. The three random controls did not show a significant difference between the mean AUC and 0.5, as expected. An AUC value of 0.5 represents a random classification test, one in which the algorithm is no better than guessing

		***p*** value of ***t*** test
1930 data	1930 random	2 · 10^−11^
1940 data	1940 random	1 · 10^−9^
1950 data	1950 random	1 · 10^−4^
0.5	1930 data	3 · 10^−14^
0.5	1940 data	4 · 10^−13^
0.5	1950 data	3 · 10^−4^
0.5	1930 random	0.1
0.5	1940 random	0.4
0.5	1950 random	0.08

Fig. 1 and Table 3 also show that the AUC (area under the curve of the receiver operating characteristic curve) for the XGBoost classification model was about 0.51, but still significantly greater than 0.50. A classification model with an AUC of 0.51 is just slightly better than guessing.

**Table 3:** The mean and standard deviation of the area under the curve of the receiver operating characteristic curve was recorded after each of the 100 different XGBoost classification models. Each run used a different set of people who did not have a severe reaction to COVID-19. The mean AUC for all three datasets was well described by a normal distribution, as confirmed by a Shapiro normality test

	Mean AUC	SD AUC
1930 data	0.515	0.017
1940 data	0.516	0.019
1950 data	0.511	0.030

## Discussion

The two conclusions of this study are divergent. First, a genetic difference exists between those who have the most severe course of COVID-19 and the general population. Second, we were not able to exploit this difference to develop a clinically useful test to distinguish between people who will experience a severe course of the disease and those who will not. We could only demonstrate a genetic risk test with an AUC of 0.51, just slightly above 0.50 which represents random guessing.

Although the AUC we found here is too low to be clinically useful, several avenues for improving the AUC exist. We were constrained by the data available to compare those who had a severe reaction to COVID-19 with the general population, but the general population probably contains a substantial number of people who would also have a severe reaction to COVID-19. A better approach would be to compare those who had a severe reaction to COVID-19 with those who were asymptomatic or had a mild reaction. Simply having a much larger number of patients who had a severe reaction might also lead to an increase in AUC.

Changes in our feature selection and classification algorithm might also improve the AUC. Our feature selection algorithm that transformed “l2r” data into our final chromosomal-scale length variation data took averages over each quarter of a chromosome. We could instead include smaller chromosome segments. Generally, we need the number of features to be much less than the number of observations (patients). So, an increase in the number of observations would allow an increase in the number of features. Also, an alternative machine learning algorithm might improve the AUC. Different algorithms perform differently on different classes of problems and XGBoost generally performs well on tabular data [8]. We did a brief test of different algorithms before choosing XGBoost as the best solution for this problem. But, for instance, a deep learning algorithm might have better performance with proper tuning.

Our results add to the recent work done by others on the link between genetics and severity of COVID-19. For instance, one study from the Netherlands identified four young men from two different families who had severe symptoms of COVID-19 and no preexisting medical conditions. Detailed genetic studies revealed that these four men all had a rare loss of function variant of TLR7, which lies on the X-chromosome [9].

A detailed study of this UK Biobank COVID-19 dataset found that Black and Asian patients were at a significantly higher risk of testing positive compared to white patients [10]. This study also attempted to derive a polygenic risk score. However, when they applied the polygenic risk score to a hold-out group, they found that the mean score was indistinguishable between the group of people who had tested positive and the group that had no positive test. In comparison, our work found that these two groups are distinguishable with a genetic risk score, but only very slightly. We measured the AUC at 0.51. They [10] do not report an AUC, but an indistinguishable test is the equivalent of an AUC of 0.50.

Other more comprehensive metastudies have identified one specific genetic component behind the severity of COVID-19. For instance, one study of COVID-19 patients who experienced respiratory failure at seven hospitals in Italy and Spain found a fairly strong association in a cluster of genes lying on part of chromosome 3 and a borderline association in chromosome 9 encompassing the ABO blood group locus [11]. The “ANA_B2” June 2020 results posted by the COVID-19 Host Genetics Initiative [12, 13] also indicate a strong association in chromosome 3 but fail to reproduce the association in chromosome 9. The COVID-19 Host Genetics Initiative “ANA_B2” study compares hospitalized COVID-19 patients to the general population and are mostly derived from patients in Europe and Brazil. Neither study attempted to derive a genetic risk score.

This study has several weaknesses. First, we cannot attribute the severity of COVID-19 to particular genetic variants. This study only finds correlations and does not establish a cause and effect. Second, while it is possible that these correlations relate to underlying biology, it is also possible that the correlations are related to ancestral differences that translate to socio-economic differences. COVID-19 severity is known to be correlated with racial/ethnic background [14, 15]. The small effect that we measured might be simply due to the larger complex effect of racial/ethnic disparities in COVID-19 severity.

## Conclusion

In conclusion, we found a significant difference exists between the structural genomics of those patients in the UK Biobank who had a severe reaction to the SARS-CoV-2 virus and the general UK Biobank population. However, a test based upon this difference would not be clinically useful in its present state since it had an AUC of 0.51.

## Data Availability

The datasets analyzed during the current study are available from UK Biobank at https://www.ukbiobank.ac.uk/
